# Transfer in Learning New Vocabulary: Memorization and Abstraction

**DOI:** 10.3390/bs15111560

**Published:** 2025-11-14

**Authors:** James A. Kole, Anna C. Johnson

**Affiliations:** School of Psychological Sciences, College of Education and Behavioral Sciences, University of Northern Colorado, Greeley, CO 80639, USA; anna.johnson@aims.edu

**Keywords:** transfer of learning, generalization, abstraction, vocabulary acquisition, word root knowledge

## Abstract

An experiment was conducted to examine whether knowledge of word meanings enables learners to infer the meanings of related words, and whether such transfer is based on memory for related exemplars or for abstract knowledge. Participants completed a word root learning task in which they learned definitions of several English words derived from a shared root (e.g., ambler, noctambulant). At an immediate test, they were assessed on definitions of studied words, new unstudied derivatives (e.g., ambulate), and word roots (e.g., ambul). A multiple regression analysis showed that accuracy on word roots, but not on studied words, predicted performance on new derivatives. These results suggest that transfer of learning was based primarily on more abstract knowledge of word root meanings rather than on memory for specific words. These findings provide novel evidence that learners can apply root-based knowledge to new word forms, and are consistent with theories proposing that transfer is supported by abstract representations.

## 1. Introduction 

Transfer, the ability to apply previously acquired knowledge to new situations (Balter & Raymond, 2023), is one of the most central concepts in the psychology of learning and memory. The importance of transfer is due to the fact that all behaviors may be viewed to some extent as reflecting transfer, as the conditions under which individuals learn knowledge are never exactly the same as those when they have to apply that knowledge. As Deese (1958) and McDaniel (2007) emphasized, nearly all education is built on the premise that learners can generalize what they have learned to novel contexts. Whether solving a real-world problem, interpreting new information, or applying skills beyond the original learning context, transfer is a fundamental indicator of meaningful learning. Yet, despite its importance, transfer is often limited. A robust literature shows that even subtle changes to stimuli, responses, task procedures, or context can dramatically reduce performance on transfer tasks (e.g., Bourne et al., 2005; Healy et al., 2006; Kole et al., 2010). This specificity of learning raises critical questions about when and how transfer occurs, and the underlying mechanisms that support it.

### 1.1. Theories of Transfer

Several theories have been proposed to explain why transfer sometimes succeeds and often fails. One of the earliest was Thorndike’s (1905) Identical Elements model, which posits that transfer depends on the overlap in elements (such as stimuli or responses) between learning and transfer tasks, with greater similarity yielding greater transfer. A similar idea underlies exemplar-based models of learning (e.g., Medin & Schaffer, 1978; Nosofsky, 1986), which propose that learners store individual examples in memory and assess new examples by comparing them to these previously encountered instances. These models have successfully predicted performance in categorization and memory tasks, emphasizing that transfer is guided by the retrieval of stored exemplars.

In contrast, abstraction-based models focus on extracting regularities across items, with transfer arising from these more abstract representations. Fuzzy Trace Theory (Brainerd & Reyna, 1990, 2015) argues that learners construct two types of memory representations: verbatim traces and gist traces. Verbatim traces capture specific and detailed information, whereas gist traces capture the essential meaning or understanding of information. By this theory, gist representations support transfer by capturing deeper conceptual meaning. Similarly, rule-based and structure-mapping theories (e.g., Gentner, 1983; Gick & Holyoak, 1980) propose that learners extract relational patterns or commonalities across examples, enabling broader generalization. These abstraction-based theories suggest that successful transfer depends on isolating the essential structure of a task, not on memory for specific examples or instances. Thus, both exemplar-based and abstraction-based mechanisms can support transfer, but they differ in whether transfer is based on memory for specific items or on a more abstract memory representation that is formed across exposure to multiple exemplars. Few paradigms, however, allow researchers to disentangle these two contributions, memory for exemplars and for abstract representations, within a single task.

### 1.2. Exemplars vs. Abstract Representations

An important challenge in transfer research is differentiating between exemplar- and abstraction-based theories of transfer, determining whether successful performance on new problems reflects memory for specific examples encountered previously or the formation of an abstract representation. An influential demonstration of abstraction-based transfer comes from Brown and Siegler’s seeding paradigm (Brown & Siegler, 1996, 2001; Groß et al., 2024; LaVoie et al., 2002; Wohldmann & Healy, 2020). In the original paradigm, participants provided population estimates for numerous countries. Following this pre-test, participants were trained using a memory seeding procedure; for the memory seeding procedure, participants were provided with true population estimates for a subset of the countries. Following training, participants were again tested on the population estimates for all countries, both immediately and after a 4-month retention interval. The results from these studies show that following the seeding procedure, population estimates were more accurate, even for countries that were not specifically studied during training, which reflects transfer. Interestingly, when comparing the immediate test to the delayed test, accuracy for seed countries decreased, indicating forgetting of a specific country’s populations, whereas accuracy for transfer countries did not (Brown & Siegler, 1996). These results suggested that participants abstracted a general metric framework during training (e.g., the average population size and standard deviation of population values), and that this abstracted information was used to estimate populations for transfer countries, rather than memory for specific populations. Follow-up work (LaVoie et al., 2002) confirmed that seeding supports both durable exemplar memory and abstracted metric knowledge.

Although studies using the seeding paradigm suggest that abstraction underlies transfer, it is also possible that individuals can use a combination of exemplar and abstraction-based strategies on different trials within the same task (e.g., Bourne et al., 2006). More recently, research has focused on individual differences in memorizing exemplars and forming abstract representations (e.g., Herzog & Goldwater, 2025; Little & McDaniel, 2015; McDaniel et al., 2014). In agreement with the findings of Brown and Siegler (1996), these studies generally find that those who form more abstract representations perform better on transfer tests (e.g., Frey et al., 2017).

### 1.3. Vocabulary Learning

One domain where the tension between memorization and abstraction is especially relevant is language. A central puzzle in language acquisition is how individuals acquire word meanings from limited input and feedback. Quine’s (1960) inscrutability of reference highlights the difficulty of identifying a word’s meaning based solely on observable use. Morphological structure, particularly the presence of word roots, provides a partial answer. More specifically, learners may infer the meanings of novel words by recognizing familiar morphemes. Experimental studies suggest that both children and adults are capable of using morphemes to infer word meanings in artificial language contexts (e.g., Behzadnia et al., 2024; Solaja & Crepaldi, 2024), suggesting that morphological cues support transfer. For example, Behzadnia et al. (2024) found that adults inferred that a novel root like torb, embedded in a word like torbla, carried a consistent meaning regardless of surface differences (e.g., torbnel, torbilm), which suggests the abstraction of root meaning. Similarly, Solaja and Crepaldi (2024) demonstrated that morphologically structured novel words were learned and recognized more effectively than non-morphemic alternatives. Even children show this pattern: Raudszus et al. (2021) found that fifth-grade readers often relied on morphological cues, sometimes even more than context, to infer the meaning of unfamiliar words. However, it remains unclear whether such transfer is driven by the abstraction of morphological information, such as root meanings, or by memory for specific exemplars. The present study addresses this gap by using a design that independently assesses both exemplar memory and abstraction.

Although theories of transfer and abstraction are well developed, many transfer tasks, such as the seeding paradigm (Brown & Siegler, 1996, 2001; Groß et al., 2024; LaVoie et al., 2002; Wohldmann & Healy, 2020), do not allow for independent measurement of memory for exemplars and abstracted representations within a single task. Moreover, some recent studies investigating morphological abstraction have used artificial language stimuli or nonwords (e.g., Behzadnia et al., 2024; Solaja & Crepaldi, 2024), which is valuable for experimental control, but may limit generalizability to real-world vocabulary learning. What is needed is a paradigm that simultaneously (1) allows for abstraction of shared structure, (2) enables memory for specific exemplars, and (3) provides measurable indices of both exemplar memory and abstraction.

### 1.4. Current Study

The present study introduces a novel vocabulary learning paradigm designed to meet these criteria, inspired by the seeding paradigm of Brown and Siegler (1996, 2001). For this paradigm, participants learned sets of words that shared common roots and then were tested on their ability to define studied words, unstudied words from the same roots, and the word roots themselves. Critically, the test structure permits independent measurement of memory for exemplars (studied words) as well as knowledge of more abstract word root meaning. This dual assessment enables us to evaluate the relative contributions of exemplar-based and abstraction-based transfer within a single task. In doing so, the study advances both theoretical models of transfer and applied understanding of vocabulary generalization.

### 1.5. Hypotheses

We hypothesized that on the immediate test, participants would show higher accuracy on definitions of studied words, indicating retention of studied exemplars, compared to new, unstudied words and word roots. We further expected that performance on unstudied words from the same roots would improve relative to a baseline measure of accuracy, indicating transfer of learning.

If exemplar memory supports transfer, then accuracy on new words should correlate with accuracy on studied words. If abstraction supports transfer, then accuracy on new words should correlate with accuracy on word roots. It is also possible that both exemplar memory and abstract knowledge contribute to transfer.

## 2. Method

### 2.1. Participants

Participants were recruited from the introductory psychology participant pool at the University of Northern Colorado. Sixty-seven students participated in the study; however, the data from 23 participants were excluded due to failure to complete the experiment, typically because they exited early or experienced technical issues. As the study was conducted online, such attrition is common in unsupervised web-based experiments at this institution. Of the remaining 44 participants, 30 identified as female and 14 as male. Participants received course credit for completing the experiment. This study was reviewed and approved by the university’s Institutional Review Board (IRB; Protocol #2005002825).

A power analysis indicated that a sample size of 28–34 participants would be required to detect a medium effect size (f = 0.25) in a repeated-measures ANOVA with three levels, assuming α = 0.05 and power = 0.80. The final sample size of 44 thus exceeded this threshold.

### 2.2. Word Root Abstraction Task

The word root abstraction task was designed to be similar to the seeding paradigm (Brown & Siegler, 1996, 2001; LaVoie et al., 2002). For this task, five word roots were selected, and five derivatives were chosen for each root, yielding a total of 25 words. An example set included the root *ambul* (meaning “move”) with derivatives such as amble, ambler, noctambulant, preamble, and ambulate. Word roots and derivatives were selected based on their potential familiarity (or lack thereof) to college students, with a goal of providing both learning opportunity and challenge. Definitions were written to avoid tautological cues (e.g., ambulate was defined as “to go from place to place” rather than using the word “move”). See Appendix A for the full set of word roots and derivatives.

Of the five derivatives for each word root, four were selected for the learning set and one was reserved for the immediate test, yielding 20 studied words and 5 unstudied derivatives used only at the test. The fifth derivative was intentionally selected to be more difficult and was not counterbalanced across participants, allowing it to serve consistently as a transfer item.

There were three phases for this task: a pre-test, a learning phase, and an immediate test.

### 2.3. Pre-Test

Participants were asked to define all 25 words prior to the learning phase. The definitions were scored dichotomously (0 = incorrect, 1 = correct) to establish a baseline for each participant’s pre-existing knowledge of studied and unstudied words.

### 2.4. Learning Phase

The learning phase followed the pre-test, during which participants studied 20 of the 25 derivatives (4 per word root). On each learning trial, a word was presented on the computer screen along with its definition, one at a time, for 6 s. After 5 words had been presented (one derivative word from each word root), participants were tested on them. They were presented with the word and a text box, and they were instructed to type the definition into the text box. This study–test cycle continued until all 20 words were presented and tested. The presentation and testing of the 20 words constituted one learning round, and participants completed 4 learning rounds such that each of the 20 words was presented and tested 4 times.

### 2.5. Immediate Test

Directly following the learning phase was an immediate test, during which participants were tested over all 20 studied words, as well as the 5 unstudied derivatives (one per root) and the 5 word roots themselves. Instead of recall, as in the pre-test and learning phase, participants were given a multiple-choice test in which each item consisted of a word (or word root) and 4 alternatives. The distractor definitions came from other word derivatives from the same root. Thus, participants were tested over three types of items: (a) 20 studied words (old words), which is a measure of retention for specific exemplars; (b) 5 unstudied words (new words), which is a measure of transfer; and (c) 5 word roots, which is a measure of abstract knowledge.

### 2.6. Analyses

The primary dependent variable was accuracy, operationalized as the proportion of correct definitions.

For the pre-test, descriptive statistics are provided, and accuracy for old words (the 20 words studied during the learning phase) was compared to new words (the 5 unstudied words presented only during the immediate test).

For the learning phase, each of 20 words (four words per word root) was presented and tested once during each learning round. Thus, the average proportion correct was computed for each of the four learning rounds. The learning phase was analyzed with a repeated-measures ANOVA, including the variable of learning round (Round 1–Round 4).

For the immediate test, participants were tested over old words, which included the 20 words from the learning set, new words, which included the 5 unstudied words that were included only during the immediate test, as well as word roots, which included the 5 word roots from which the old and new words were derived. The average proportion correct was calculated separately for old words, new words, and word roots; the immediate test was analyzed with a repeated-measures ANOVA, including the variable of item type (old words, new words, word roots). Planned comparisons were conducted between each item type to assess pairwise differences in performance. A second 2 × 2 repeated-measures ANOVA was also conducted on the immediate test data. For this analysis, the data for word roots were excluded; the analysis included the variables of test (pre-test, immediate test) and word type (old, new). This analysis allows for the comparison of old words to new words, as well as an examination of whether increases in accuracy from pre-test to immediate test differed for the two word types.

To investigate mechanisms underlying transfer during the immediate test, a multiple regression was conducted that included accuracy on new words as the dependent variable, and accuracy for old words, for word roots, and pre-test accuracy for new words as predictor variables.

## 3. Results

### 3.1. Pre-Test

For the 20 words that would be studied during the learning phase (old words), the average number of correct definitions was 0.403 (*SD* = 0.225), indicating that, at the start of the experiment, participants knew less than half of the definitions of the words in the learning set. The second average, for the 5 words that served as new words during the immediate test, was 0.200 (*SD* = 0.202), indicating that participants knew a fifth of the new words at the start of the study. These words were intentionally selected a priori to be more difficult or lesser known by participants to avoid ceiling effects on the immediate test. The difference between old and new words was significant (*F*(1, 43) = 22.482, *MSE* = 0.040, *p* < 0.001, *η_p_*^2^ = 0.343).

### 3.2. Learning Phase

**Figure 1:**
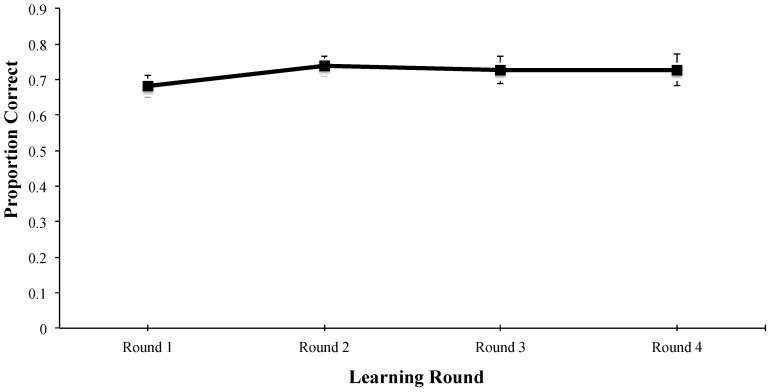
Proportion correct during the learning phase as a function of learning round. Note. Error bars represent standard errors of the mean. Alt text. Accuracy during the learning phase was steady across learning rounds.

The analysis of the learning phase revealed that the main effect of learning round was not significant (*F*(3, 129) = 2.111, *MSE* = 0.013, *p* = 0.102, *η_p_*^2^ = 0.047). As evident in Figure 1, accuracy was fairly high at the start of the learning phase and remained so throughout. The lack of improvement is likely due to the fact that a study–test procedure was used for the learning phase; that is, participants saw the correct definitions of the words first and were then tested on them afterwards, and only 5 words were presented and tested at a time, some of which were already known according to the pre-test results.

### 3.3. Immediate Test

**Figure 2:**
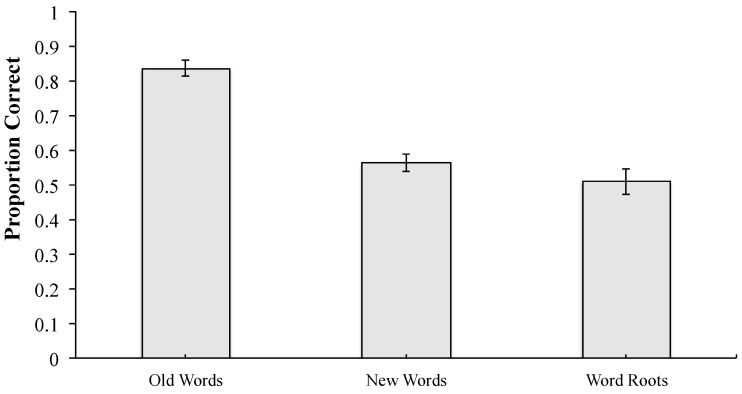
Proportion correct during the immediate test as a function of item type. Note. Error bars represent standard errors of the mean. Alt text. Accuracy on the immediate test was significantly higher for old words than for new words and word roots; accuracy did not differ between new words and word roots.

The analysis of proportion correct on the immediate test revealed a main effect of item type (*F*(2, 86) = 46.761, *MSE* = 0.029, *p* < 0.001, *η_p_*^2^ = 0.521). As shown in Figure 2, proportion correct was highest for old words presented during the learning phase, and lower for both new words and word roots. Planned comparisons revealed that the differences between old words and new words (*p* < 0.001) and between old words and word roots (*p* < 0.001) were both significant. The difference between new words and word roots was not significant (*p* = 0.077).

**Figure 3:**
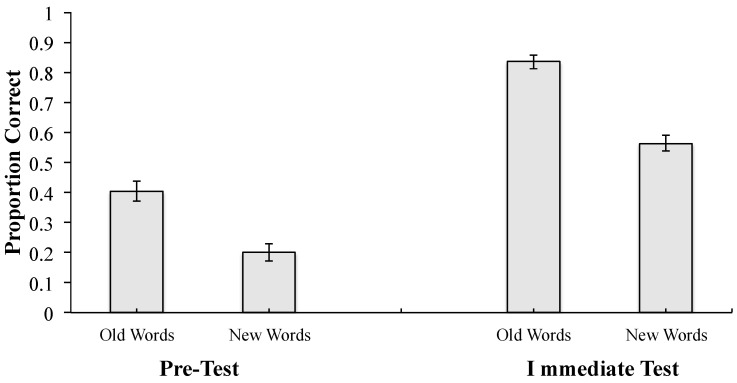
Proportion correct as a function of test and word type. Note. Error bars represent standard errors of the mean. Alt text. Accuracy increased significantly from the pre-test to the immediate test, but the interaction between test and word type was not significant, suggesting equivalent gains for old and new items.

Accuracy for old words might have been higher than for new words at the immediate test simply because participants knew more definitions of old words at the start of the experiment, as reflected in the pre-test scores. To directly examine whether learning gains differed for new and old words, a 2 (Word Type: Old, New) × 2 (Test: Pre-Test, Immediate Test) repeated-measures ANOVA was conducted. This analysis revealed a significant main effect of test, *F*(1, 43) = 202.25, *MSE* = 0.034, *p* < 0.001, *η_p_*^2^ = 0.825, with accuracy increasing from pre-test (*M* = 0.302, *SD* = 0.160) to immediate test (*M* = 0.699, *SD* = 0.108). There was also a significant main effect of word type (*F*(1, 43) = 62.02, *MSE* = 0.040, *p* < 0.001, *η_p_*^2^ = 0.591) with higher overall accuracy for old words (*M* = 0.619, *SD* = 0.136) than for new words (*M* = 0.382, *SD* = 0.147). Critically, the interaction between word type and test was not significant (*F*(1, 43) = 1.68, *MSE* = 0.030, *p* = 0.202, *η_p_*^2^ = 0.038), indicating that the increase in accuracy from the pre-test to the immediate test did not differ significantly between new and old words (see Figure 3).

Thus, although accuracy overall was higher for old words than for new words, the absence of an interaction suggests that gains over baseline were statistically equivalent for old and new items, even though the new items were never directly presented during the learning phase. This supports the interpretation that participants generalized their knowledge to the new, unstudied words, consistent with transfer of learning.

To explore the mechanisms underlying transfer, a multiple regression analysis was conducted to determine whether performance on new words reflected retention of studied words or knowledge of more abstract word root meanings. Accuracy for new words served as the dependent variable, and accuracy for old words, word roots, and pre-test accuracy for new words were included as predictors. The overall model was significant (*F*(3, 40) = 12.17, *p* < 0.001, *R*^2^ = 0.48), indicating that nearly half of the variance in new word performance was accounted for by the predictors (see Table 1). Tests of individual predictors revealed that accuracy for old words did not significantly predict new word performance (*t* = −1.70, *p* = 0.098, *sr* = −0.194, *sr*^2^ = 0.04), suggesting that memory for studied words alone did not account for the ability to infer definitions of new words. In contrast, accuracy for word roots significantly predicted new word accuracy (*t* = 5.68, *p* < 0.001, *sr* = 0.649, *sr*^2^ = 0.42), uniquely accounting for 42% of the variance in new word accuracy beyond the other predictors. This finding is more consistent with abstraction-based models of transfer.

## 4. General Discussion

The present study investigated whether memory for exemplars or for more abstract knowledge supports generalization to novel words. Participants studied sets of words that shared common word roots, and were later tested on both previously studied words and novel derivatives of those word roots. Gains in accuracy were statistically equivalent for studied (old) and unstudied (new) words, despite the fact that the new words were never presented during the learning phase. This pattern suggests that participants were not simply recalling studied items but were generalizing to novel derivatives, thereby demonstrating transfer.

To better understand the mechanisms behind this generalization, we conducted a regression analysis predicting novel word performance from multiple factors. The analysis revealed that accuracy for old words (an index of retention or exemplar memory) did not reliably predict performance on new items, suggesting that exemplar memory did not contribute to transfer. In contrast, accuracy for word roots explained a substantial and unique portion of variance in new word performance. This result suggests that more abstract knowledge appears to support transfer, enabling learners to infer the meanings of new words they had never directly encountered.

These results are consistent with studies utilizing the seeding paradigm (Brown & Siegler, 1996), which also concluded that abstract representations facilitate transfer. More broadly, these findings align with abstraction-based theories of transfer, such as Fuzzy Trace Theory (Brainerd & Reyna, 1990, 2015) and structure-mapping theory (Gentner, 1983), which posit that transfer depends on gist-level or relational understanding rather than memory for specific exemplars.

This study contributes to our understanding of how learners overcome the inscrutability of reference (Quine, 1960) problem in language learning by showing that participants were able to infer the meanings of novel words based on structural cues embedded in related words. Despite never studying some derivatives directly, their performance suggests they may have accessed more abstract morphological knowledge to support generalization. These results build on recent studies demonstrating that learners, both children and adults, can use morphemic cues to generalize meaning (e.g., Behzadnia et al., 2024; Raudszus et al., 2021; Solaja & Crepaldi, 2024) but extend that work by isolating abstraction from exemplar-based memory. The word root abstraction task introduced here offers a novel method for disentangling these mechanisms and may serve as a valuable tool for studying semantic generalization, morphological processing, and individual differences in language learning.

Although the regression results suggest that abstract morphological knowledge played the primary role in the transfer of learning to unstudied words, it is possible that multiple strategies contributed to performance, either between or within participants. That is, some participants may have relied more on specific studied exemplars, while others inferred word meaning through root-based abstraction; such a possibility is consistent with the abstractor–memorizer distinction (e.g., Herzog & Goldwater, 2025; Little & McDaniel, 2015; McDaniel et al., 2014). Alternatively, participants may have used a combination of strategies across different items (e.g., Bourne et al., 2006). Future work could explore these possibilities by tracking item-level response patterns or incorporating strategy reports to better understand individual differences in transfer mechanisms.

The ability to extract meaning from structural cues aligns with broader proposals that learners use grammatical patterns to infer word meaning, such as in syntactic bootstrapping. While our study focused on word roots and morphology, this mechanism shares theoretical overlap with syntactic bootstrapping, the idea that learners can use morphosyntactic information to infer the meanings of new words (Gleitman, 1990; Babineau et al., 2024). Syntactic bootstrapping typically emphasizes how affixes or sentence structure constrain possible meanings of verbs or nouns. Our findings suggest that structural inference may also apply at the word root level. Bridging these literatures could enrich theories of word learning by highlighting the multiple levels of linguistic structure that learners exploit.

Several limitations should be acknowledged. First, the word roots used in this study may have differed in familiarity or morphological transparency. Future research could systematically examine the roles of root regularity, word frequency, and individual vocabulary knowledge in supporting abstraction. Second, the task was administered under tightly controlled experimental conditions. Applying this paradigm in more naturalistic settings, such as classrooms or second-language learning environments, could reveal how abstraction operates in real-world contexts. Third, the learning phase used an interleaved design, in which items from different word roots were mixed during study. Although prior work suggests that interleaving supports abstraction and transfer (e.g., Kornell & Bjork, 2008), some recent findings suggest that blocked conditions may provide even stronger support for rule learning, particularly when regularities must be extracted across similar items (e.g., Little & Nepangue, 2025; Noh et al., 2016).

A fourth limitation is that word root knowledge was not assessed at pre-test, so it is possible that some participants possessed prior knowledge of certain roots. Future research might test younger or less metalinguistically aware populations, such as children or early readers, who may lack this knowledge. However, several factors suggest that abstract morphological knowledge, whether newly acquired or pre-existing, underlies the observed transfer. Accuracy on new words was predicted by root knowledge even after controlling for pre-test accuracy and memory for studied words, and performance on word root items remained moderate, suggesting incomplete prior knowledge. Thus, the findings still support the conclusion that abstract representations, rather than exemplar memory, facilitate generalization. Finally, incorporating additional outcome measures such as delayed tests, sentence-level generalization, or broader retention metrics could further validate this method as a tool for assessing verbal abstraction and transfer.

Fifth, this paradigm provides partial ecological validity by using real English words and a vocabulary learning format, which are relevant to educational settings. However, the isolated word presentation without sentence context limits the extent to which results generalize to natural reading or language use.

Beyond theoretical contributions, the findings have practical implications for language learning and educational practice. The ability to abstract structural features such as word roots may facilitate vocabulary growth, especially when learners encounter unfamiliar words in context. Teaching strategies that emphasize root meaning, morphological analysis, and pattern recognition may help students infer new word meanings independently, supporting long-term word learning. Additionally, the word root abstraction task may serve as a useful diagnostic tool for identifying learners who struggle with generalization or who rely heavily on memorization, which may impair transfer. More broadly, this work highlights the value of designing instructional experiences that promote abstraction.

## Figures and Tables

**Table 1:** Regression Coefficients for the Multiple Regression Model.

Variable	*B*	β	*SE*	*t*	*p* Value	*sr* ^2^
Constant	0.466	--	0.119	3.914	<0.001	--
Old Word Accuracy	−0.225	−0.196	0.133	−1.695	0.098	0.04
Word Root Accuracy	0.468	0.660	0.082	5.675	<0.001	0.42
Pre-Test Accuracy	0.241	0.280	0.099	2.422	0.020	0.07

## Data Availability

The data presented in this study are available from the corresponding author upon reasonable request.

## Appendix A. Word Roots and Derivatives

Note: Italicized items served as new words on the immediate test.

ambul: move

amble: meander without urgency

ambler: a person who walks slowly

noctambulant: pertaining to sleepwalking

preamble: introductory remarks

*ambulate: to go from place to place*

bene: good

benefactor: kindly helper

beneficial: advantageous

benevolent: charitable

benediction: a form of blessings

*beneficent: generous*

pan: all

panorama: a wide view

pantheism: a belief in God as nature/the universe

pandemic: a prevalent, wide-sweeping disease

panoply: a magnificent display

*panacea: something that cures everything*

per: through

permeate: saturate

perennial: everlasting

permanent: forever

persist: to continue steadfastly

*peregrinate: to walk or travel; especially, on foot*

spir: breathe

inspire: influence positively

perspire: to sweat through pores

spirit: the vital principal in humans which animates

respirometer: measures rate of oxygen consumption

*aspiration: supply with air*
